# Evaluating Risk Factors for Surgical Site Occurrences: Infection and Wound Dehiscence Post Definitive Surgery for Sacrococcygeal Pilonidal Sinus Disease

**DOI:** 10.7759/cureus.97368

**Published:** 2025-11-20

**Authors:** Ellen G Maclean, Mary M Teoh, Cian Casey, Eoghan Blount, David Walsh, Alexander Armanios, Munyaradzi G Nyandoro

**Affiliations:** 1 General and Colorectal Surgery, Fiona Stanley Hospital, Perth, AUS; 2 General and Colorectal Surgery, Sir Charles Gairdner Hospital, Perth, AUS; 3 General Surgery, Fiona Stanley Hospital, Perth, AUS; 4 General Surgery, Fremantle Hospital, Perth, AUS; 5 School of Medicine, The University of Western Australia, Perth, AUS

**Keywords:** definitive surgery, sacrococcygeal pilonidal disease, surgical site infection, surgical site occurrences, wound dehiscence

## Abstract

Background: Sacrococcygeal pilonidal sinus disease (SPD) predominantly affects young adults and is associated with high morbidity after surgery. Surgical site infections (SSIs) and wound dehiscence (WD) are frequent and clinically significant complications. This study evaluated their rates and identified associated risk factors across multiple surgical techniques.

Methods: A retrospective multi-centre cohort study was conducted across eight hospitals in Western Australia (2010-2019). Patients aged ≥15 years undergoing elective definitive SPD surgery with flap or secondary intention techniques were included. Data were extracted from medical records and electronic databases. SSI risk was analysed with univariate and multivariate logistic regression, while WD was examined using Cox proportional hazards modelling. Outcomes included 30-day re-presentation and readmission rates, with extended follow-up for recurrence.

Results: A total of 774 patients were analysed. SSIs occurred in 28.8% and WD in 28.4%. The cohort included 79.7% male patients, with a mean age of 27.08 (SD±9.06) years and a BMI of 28.58 (SD±6.15).

Multivariate analysis demonstrated that secondary intention techniques (OR 6.0, p<0.01), other flap procedures (OR 3.1, p=0.03), overweight status (OR 1.9, p=0.01), and WD (OR 50.6, p<0.01) were independent risk factors for SSIs. In contrast, clear surgical margins and methylene blue use were protective.

Cox regression showed increased WD risk with Karydakis flap (HR 1.8, p=0.04), modified Limberg flap (HR 2.0, p=0.03), and smoking (HR 1.4, p=0.04). Within 30 days, 27% re-presented and 7.5% were readmitted, mainly for SSIs or WD, with regional variation reflecting practice differences.

Conclusion: SSIs and WD remain common and burdensome after SPD surgery. The modified Karydakis flap demonstrated the most favourable risk profile. Optimising patient factors, meticulous surgical technique, and structured training are essential to reduce complications and improve outcomes.

## Introduction

Sacrococcygeal pilonidal sinus disease (SPD), first described by Mayo in 1833, predominantly affects young men and has experienced a notable increase in incidence over the last two decades [1-8]. The term ‘pilonidal’, derived from the Latin words pilus (hair) and nidus (nest), was introduced by Hodges in 1880 [6]. SPD is considered an acquired disease originating in the hair follicles of the natal cleft in the sacrococcygeal region. Established risk factors include male sex, hirsutism, obesity, a deep gluteal cleft, and a sedentary lifestyle [1-4,6-11]. A range of surgical techniques has been developed for the management of SPD, although no consensus exists regarding a gold standard procedure. This lack of agreement arises from high complication rates, including recurrence, surgical site infections (SSIs), wound dehiscence (WD), haematoma, seroma, sphincter injury and flap oedema [2-4,7-14]. Current literature supports flap reconstruction techniques as primary options for chronic SPD yet provides no clear guidance on which specific flap should be used [12-16]. The ideal procedure should require minimal hospitalisation, cause little discomfort, have low recurrence rates, allow for rapid return to work or study, and impose a limited burden on the health system [2,6,9,16,17].

The purpose of this study was to evaluate the occurrence of SSIs and WD, together with their associated risk factors, following definitive surgery for chronic SPD. Both flap-based reconstruction and secondary intention procedures were assessed, with the additional aim of identifying predictors of surgical site complications that may inform future practice.

This research represents the first Australian multi-centre cohort study to investigate SSIs and WD across different surgical approaches to SPD, including both flap and secondary intention techniques. Previous studies have often been limited to single centres or individual surgeons and have not examined risk factors in detail [8,10,11,18-21]. Their applicability to the Australian context is uncertain, given differences in patient demographics and healthcare systems. By addressing these limitations, the present study contributes to a clearer understanding of current surgical practice, local disease burden and associated outcomes. These insights may help refine decision-making, reduce complication rates and improve patient outcomes in the management of this common condition.

## Materials and methods

This multi-centre observational analytical retrospective cohort study reviewed medical records from eight hospitals in Western Australia between January 2010 and December 2019. Hospitals A, B and G were part of the East Metropolitan Health Service (EMHS), C, D and F belonged to the South Metropolitan Health Service (SMHS), and E and H were within the North Metropolitan Health Service (NMHS). These sites included both tertiary teaching centres and peripheral hospitals, thereby providing a representative cross-section of surgical practice in the state. The choice of procedure at each site was influenced by surgeon preference, disease characteristics, and patient factors, thereby representing real-world clinical practice patterns. Each participating centre had fellowship-trained surgeons with substantial operative experience, ensuring uniformity in procedural expertise and minimising inter-centre variability. 

**Figure 1:**
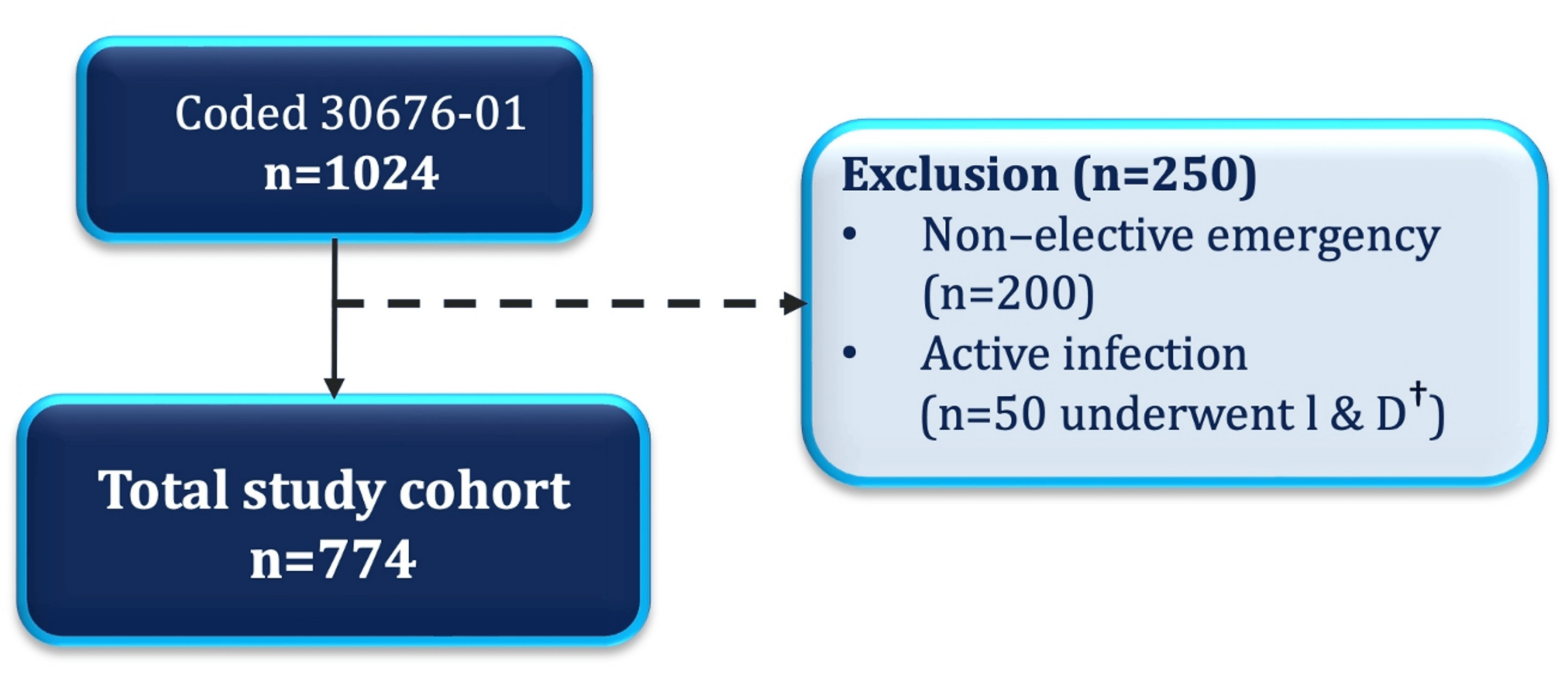
Case selection ^ †^I & D = Incision and drainage of an acute abscess

Eligible participants were patients aged 15 years or older who underwent elective definitive surgery for chronic SPD. Both flap-based reconstruction and procedures managed by secondary intention, including laying open with marsupialisation, were included. Cases were identified through the Western Australian health databases TOPAS, WebPAS and iSOFT using ICD-10-AM codes and the procedure codes (30676-01) for excision or marsupialisation of a pilonidal cyst. Patients were excluded if surgery was performed as an emergency or if active infection with an abscess was present at the time of operation. Each patient was included only once, and all re-do cases in this study were patients who had their index procedures performed at centres outside the study catchment area, for which the investigators did not have access to the original procedure and follow-up details. The sample size was based on all available eligible cases during the study period. See Figure 1 for an overview of case selection.

Data were extracted by seven independent investigators (EM, MT, CC, EB, DW, AA, MGN) from medical records and cross-referenced with electronic databases to ensure accuracy. Missing data were assessed for randomness and excluded pairwise from analyses. Variables collected included demographics, comorbidities, operative details, histopathology, recurrence, and re-presentation or readmission. Information on outpatient clinic utilisation, wound care services, and hospital-in-the-home (HITH) involvement was also recorded. Thirty-day re-presentation and readmission rates were recorded. Patients were considered not to have experienced SPD recurrence if no re-admission or re-presentation with SPD was registered during the study period.

Definitions for key clinical variables were standardised prior to analysis. Hirsutism was categorised into abundant, little or none. BMI was categorised according to WHO definitions. Immediate complications referred to events occurring during the index hospital admission, whereas delayed complications were those occurring within 30 days. Recurrence was defined as reappearance of SPD beyond 30 days. SSI was defined using the Dell criteria and classified as superficial or deep. WD was defined as separation of the wound either during the index admission or at re-presentation to the hospital and wounds were classified according to the CDC surgical wound classification system.

The primary objective was to identify independent risk factors associated with SSIs following definitive surgery for SPD. The secondary objective was to evaluate predictors of WD and overall surgical site occurrences. 

Categorical variables were compared using Pearson’s chi-square test or Fisher’s exact test, as appropriate. Continuous variables were compared using Student’s t-test or Mann-Whitney U test, depending on data distribution.

Univariate analyses were performed for all candidate variables, with those achieving p<0.1 entered into multivariate logistic regression models to identify independent predictors of SSI. Cox regression models were used to analyse time-to-event data. All analyses were conducted using IBM SPSS Statistics for Windows, Version 29 (Released 2023; IBM Corp., Armonk, New York, United States). A two-tailed p-value of <0.05 was considered statistically significant.

For SSIs, univariate analyses were followed by multivariate binary logistic regression to estimate odds ratios (ORs) with 95% confidence intervals (CIs). Independent variables considered included patient characteristics (BMI, diabetes, smoking status, hirsutism, sex, and age), perioperative factors (antibiotic prophylaxis, type of skin preparation, methylene blue use, operative duration, drain use, and whether it was a redo procedure), and pathological findings (surgical margins, wound classification).

For WD, univariate analyses were followed by Cox proportional hazards regression to model time to wound separation. WD was defined as a clinically diagnosed wound separation occurring during the index admission or at hospital re-presentation. Covariates considered included the same baseline, operative and pathological variables, in addition to post-operative complications (SSI, haematoma, seroma). Hazard ratios (HRs) with 95% CIs were reported. Proportional hazards assumptions were tested, and all variables meeting significance in univariate analysis were assessed in the multivariate model.

This study was approved by the South Metropolitan Health Service Human Research Ethics Committee (RGS511) and the University of Western Australia Human Research Ethics Committee (RA/4/20/4547) as a low-risk project. A waiver of consent was granted for this retrospective arm of the overarching study as all data were fully de-identified prior to analysis, in accordance with the National Statement on Ethical Conduct in Human Research (National Health and Medical Research Council Act, NHMRC, 2018. This study adheres to the STROBE (Strengthening the Reporting of Observational Studies in Epidemiology) and STROBE-RECORD statements for cohort studies. 

## Results

Demographics

A total of 774 patients from eight hospitals were included in the study. Participant demographics are demonstrated in Table 1; most participants were male (n = 617, 79.7%) with a mean age of 27.1 years (SD 9.1). Men were slightly older than women at the time of surgery (27.3 vs 26.2 years). Body weight ranged from 32 to 210 kg, with men heavier on average than women (89.9 vs 80.5 kg). Mean height was 177.6 cm for men and 165.7 cm for women. The overall mean body mass index (BMI) was 28.6 (SD 6.2), consistent with the overweight range. More than one-third of the cohort was classified as obese (37.1%), and over half were current smokers (50.9%). The majority did not have diabetes (90.3%) and were not hirsute (73.6%) (Table 1).

**Table 1:** Participant demographics per SPD surgery types Values are the number of participants (%) unless otherwise indicated. ^*^ Pearson Chi-Square analysis, and Fisher’s exact test (for cell values <5), and * denotes significance at p<0.05 (indicated in bold) ^a^ SPD: Sacrococcygeal pilonidal sinus disease ^b^ BMI: Body mass index

Variable (N = 774)			Cohort	SPD ^a^ Surgery Procedures					
			Total Sample	KF	MKF	MLF	SIT	OFT	p-value
	Total Sample	N=	774	302	242	92	90	48	
Sex	Male	n=	617	235	189	74	77	42	0.309
		%	79.7%	77.8%	78.1%	80.4%	85.6%	87.5%	
	Female	n=	157	67	53	18	13	6	
		%	20.3%	22.2%	21.9%	19.6%	14.4%	12.5%	
BMI group ^b^	Healthy Weight	n=	221	85	73	27	25	11	0.971
		%	28.6%	28.1%	30.2%	29.3%	27.8%	22.9%	
	Overweight	n=	266	101	87	30	31	17	
		%	34.4%	33.4%	36.0%	32.6%	34.4%	35.4%	
	Obese	n=	287	116	82	35	34	20	
		%	37.1%	38.4%	33.9%	38.0%	37.8%	41.7%	
Age group	15 to 19yrs	n=	161	57	57	18	21	8	0.542
		%	20.8%	18.9%	23.6%	19.6%	23.3%	16.7%	
	20 to 29yrs	n=	371	148	103	48	44	28	
		%	47.9%	49.0%	42.6%	52.2%	48.9%	58.3%	
	30yrs +	n=	242	97	82	26	25	12	
		%	31.3%	32.1%	33.9%	28.3%	27.8%	25.0%	
Diabetes	No	n=	699	271	225	88	76	39	0.013*
		%	90.3%	89.7%	93.0%	95.7%	84.4%	81.3%	
	Yes	n=	75	31	17	4	14	9	
		%	9.7%	10.3%	7.0%	4.3%	15.6%	18.8%	
Hirsutism	No	n=	570	226	189	72	58	25	0.001*
		%	73.6%	74.8%	78.1%	78.3%	64.4%	52.1%	
	Yes	n=	204	76	53	20	32	23	
		%	26.4%	25.2%	21.9%	21.7%	35.6%	47.9%	
Smoker	No	n=	380	137	131	45	46	21	0.305
		%	49.1%	45.4%	54.1%	48.9%	51.1%	43.8%	
	Yes	n=	394	165	111	47	44	27	
		%	50.9%	54.6%	45.9%	51.1%	48.9%	56.3%	
Ex-smoker / recent <3months	No	n=	693	277	214	78	83	41	0.228
		%	89.5%	91.7%	88.4%	84.8%	92.2%	85.4%	
	Yes	n=	81	25	28	14	7	7	
		%	10.5%	8.3%	11.6%	15.2%	7.8%	14.6%	
Financial	Public	n=	721	285	219	85	88	44	0.156
		%	93.2%	94.4%	90.5%	92.4%	97.8%	91.7%	
	Private	n=	53	17	23	7	2	4	
		%	6.8%	5.6%	9.5%	7.6%	2.2%	8.3%	

Surgical procedure types

Nine distinct procedures were identified and recategorised for analysis. These included the Karydakis flap (KF, 39.0%), modified Karydakis flap (MKF, 31.3%), modified Limberg flap (MLF, 11.9%), laying open with marsupialisation as a secondary intention technique (SIT, 11.6%), and a group of less common procedures such as Bascom’s cleft lift, gluteus maximus myocutaneous flap, Z-plasty and V-Y advancement, which were grouped as other flap techniques (OFT, 6.2%). Most participants were considered public patients (93.2%), and almost half of the procedures were undertaken within the South Metropolitan region (49.1%). The hospitals contributing the largest volumes were A, B and E, together accounting for more than 60% of cases (Table 1).

Across the decade of data collection, KF and MKF were the most frequently performed procedures (Table 2). From 2013 onwards, MKF and MLF became more common, with a corresponding reduction in KF. Most operations were definitive index procedures (85.0%), with redo surgery accounting for 15.0%. SIT and OFT were more likely to be performed as secondary procedures, whereas KF, MKF and MLF were predominantly index procedures.

**Table 2:** Initial surgery details per SPD surgery type – categorical variables Values are the number of participants (%) unless otherwise indicated. ^*^ Pearson Chi-Square analysis, and Fisher’s exact test (for cell values <5), and * denotes significance at p<0.05 (indicated in bold) ^a^ SPD: Sacrococcygeal pilonidal sinus disease,^ b^ IVABs: Intravenous antibiotics

Variable (N = 774)			Cohort	SPD ^a^ Surgery Procedures					
			Total Sample	KF	MKF	MLF	SIT	OFT	p-value
	Total Sample	N=	774	302	242	92	90	48	
Re-do procedure	No	n=	658	280	214	75	63	26	< 0.001*
		%	85.0%	92.7%	88.4%	81.5%	70.0%	54.2%	
	Yes	n=	116	22	28	17	27	22	
		%	15.0%	7.3%	11.6%	18.5%	30.0%	45.8%	
IVABS on induction ^b^	No	n=	15	5	4	1	5	0	0.154
		%	1.9%	1.7%	1.7%	1.1%	5.6%	0.0%	
	Yes	n=	759	297	238	91	85	48	
		%	98.1%	98.3%	98.3%	98.9%	94.4%	100.0%	
Query infected site	No	n=	729	292	234	88	68	47	< 0.001*
		%	94.2%	96.7%	96.7%	95.7%	75.6%	97.9%	
	Yes	n=	45	10	8	4	22	1	
		%	5.8%	3.3%	3.3%	4.3%	24.4%	2.1%	
Wound classification	Clean	n=	101	25	42	14	8	12	< 0.001*
		%	13.0%	8.3%	17.4%	15.2%	8.9%	25.0%	
	Clean/ Contaminated	n=	548	234	171	63	50	30	
		%	70.8%	77.5%	70.7%	68.5%	55.6%	62.5%	
	Contaminated	n=	125	43	29	15	32	6	
		%	16.1%	14.2%	12.0%	16.3%	35.6%	12.5%	
Drain-situ	No	n=	407	160	126	23	90	8	< 0.001*
		%	52.6%	53.0%	52.1%	25.0%	100.0%	16.7%	
	Yes	n=	367	142	116	69	0	40	
		%	47.4%	47.0%	47.9%	75.0%	0.0%	83.3%	
Methylene blue used	No	n=	582	258	143	53	87	41	< 0.001*
		%	75.2%	85.4%	59.1%	57.6%	96.7%	85.4%	
	Yes	n=	192	44	99	39	3	7	
		%	24.8%	14.6%	40.9%	42.4%	3.3%	14.6%	
Skin prep type	Povidone	n=	644	256	206	70	81	31	0.002*
		%	83.2%	84.8%	85.1%	76.1%	90.0%	64.6%	
	Chlorhexidine	n=	130	46	36	22	9	17	
		%	16.8%	15.2%	14.9%	23.9%	10.0%	35.4%	

Post-operative complications

The overall SSI rate was 28.8% (n = 223). OFT recorded the highest SSI rate (47.9%), while MKF had the lowest (21.9%). SIT was also strongly associated with infection. The overall WD rate was 28.4% (n = 220). OFT (39.6%) and KF (37.1%) had the highest WD rates, whereas MKF was associated with the lowest (25.2%). As SIT heals by secondary intention, these cases were excluded from the WD sub-analysis. Haematoma occurred in 8.4% (n = 65), with SIT showing the highest rate (12.2%) and MLF the lowest (2.2%). Seroma was rare, affecting only 1.4% (n = 11). A summary of post-operative complications is provided in Table 3, with additional wound-related events outlined in Table 4.

**Table 3:** Post-operative surgery complication details per SPD surgery type Values are the number of participants (%) unless otherwise indicated. ^*^ Pearson Chi-Square analysis, and Fisher’s exact test (for cell values <5), and * denotes significance at p<0.05 (indicated in bold) ^a^ SPD: Sacrococcygeal pilonidal sinus disease ^b^ Margins - n= 653, not all specimens were sent for histopathology

Variable (N = 774)			Cohort	SPD ^a^ Surgery Procedures					
			Total Sample	KF	MKF	MLF	SIT	OFT	p-value
	Total Sample	N=	774	302	242	92	90	48	
Recurrence	No	n=	600	220	206	83	62	29	<0.001*
		%	77.5%	72.8%	85.1%	90.2%	68.9%	60.4%	
	Yes	n=	174	82	36	9	28	19	
		%	22.5%	27.2%	14.9%	9.8%	31.1%	39.6%	
Surgical site infection	No	n=	551	206	189	70	61	25	0.002*
		%	71.2%	68.2%	78.1%	76.1%	67.8%	52.1%	
	Yes	n=	223	96	53	22	29	23	
		%	28.8%	31.8%	21.9%	23.9%	32.2%	47.9%	
Dehiscence (n=684)	No	n=	464	190	181	64	-	29	<0.001*
		%	67.8%	62.9%	74.8%	69.6%	-	60.4%	
	Yes	n=	220	112	61	28	-	19	
		%	32.2%	37.1%	25.2%	30.4%	-	39.6%	
Hematoma	No	n=	709	275	222	90	79	43	0.081
		%	91.6%	91.15	91.7%	97.85	87.8%	89.6%	
	Yes	n=	65	27	20	2	11	5	
		%	8.4%	8.9%	8.3%	2.2%	12.2%	10.4%	
Seroma	No	n=	763	296	241	92	87	47	0.111
		%	98.6%	98.0%	99.6%	100%	96.7%	97.9%	
	Yes	n=	11	6	1	0	3	1	
		%	1.4%	2.0%	0.4%	0.0%	3.3%	2.1%	
Any post-op complication	No	n=	486	180	169	61	50	26	0.033*
		%	62.8%	59.6%	69.8%	66.3%	55.6%	54.2%	
	Yes	n=	288	122	73	31	40	22	
		%	37.2%	40.4%	30.2%	33.7%	44.4%	45.8%	
Clear margins^ b^	No	n=	137	63	36	10	22	6	0.014*
		%	21.0%	24.8%	17.8%	12.0%	30.6%	14.3%	
	Yes	n=	516	191	166	73	50	36	
		%	79.0%	75.2%	82.2%	88.0%	69.4%	85.7%	
Cosmetic complaints	No	n=	728	286	232	86	79	45	0.090
		%	94.1%	94.7%	95.9%	93.5%	87.8%	93.8%	
	Yes	n=	46	16	10	6	11	3	
		%	5.9%	5.3%	4.1%	6.5%	12.2%	6.3%	

**Table 4:** Other 30-day post-op complications Values are the number of participants (%) unless otherwise indicated. ^a^ Sub-analysis of the on-referrals

Variable	Number (Proportion)	
	(n)	%
Persistent wound discharge	227	29.3
Referred to other specialties	29	3.7
Plastics	24	82.8 ^a^
Wound hypergranulation	40	5.2
Fistula	11	1.4
Seroma	11	1.4
Sphincter damage	8	1.0
30-day mortality	0	0.0

Risk factors for surgical site infection

**Figure 2:**
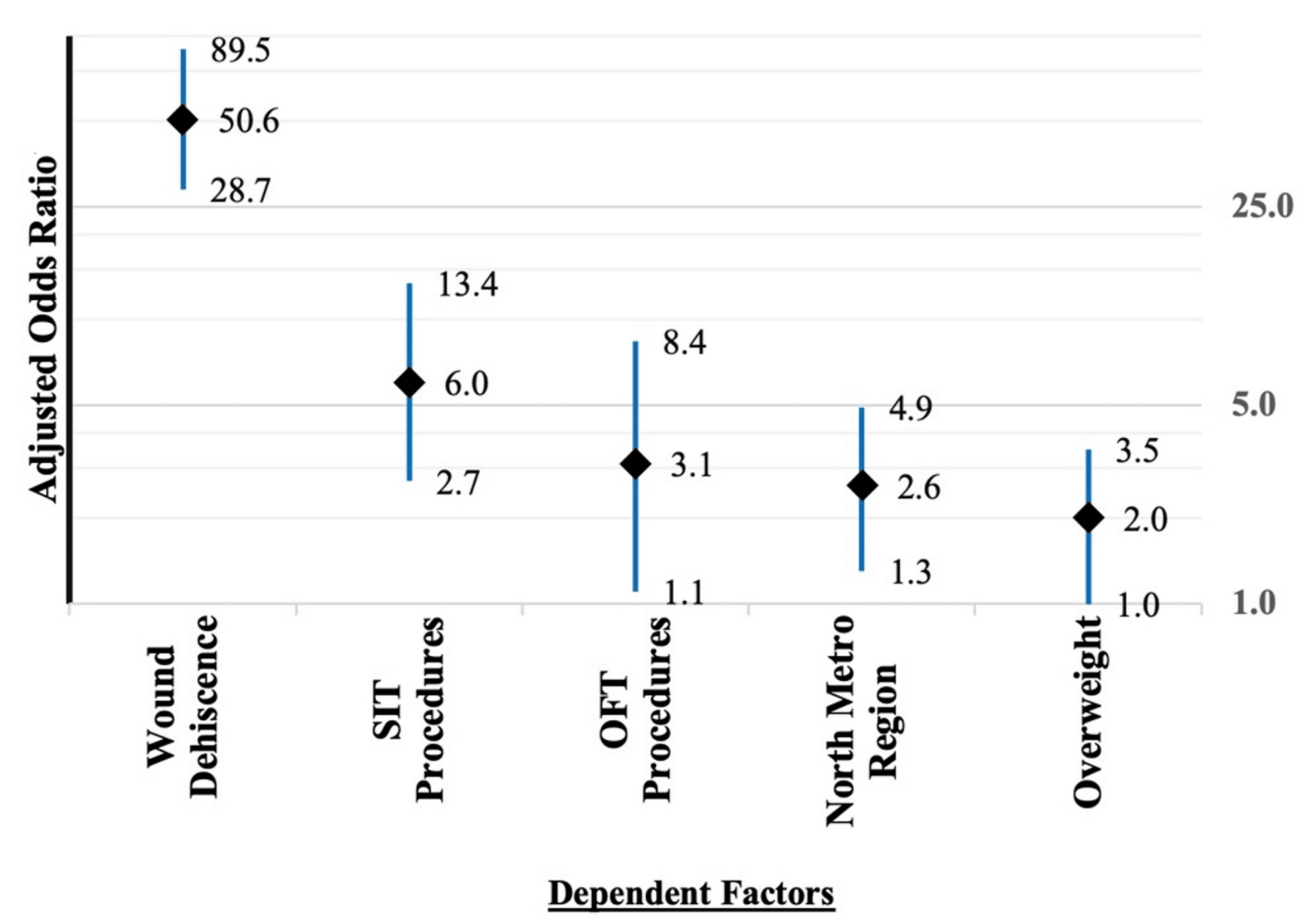
Multivariate logistic regression (N=774) Graphic logarithmic representation with the likelihood of surgical site infection as the dependent variable SIT: Secondary Intention Techniques; OFT: Other Flap Techniques

Univariate analysis demonstrated significant associations between SSI and several factors, including OFT procedures, absence of methylene blue staining, use of chlorhexidine skin preparation, operations performed in areas suspected of infection, diabetes, smoking, hirsutism and positive surgical margins. Univariate analysis, with the likelihood of SSI as the dependent variable, is presented in Table 5. However, and surprisingly, there was no significant association of SSI with obesity or re-do procedures, but they were trending towards significance (p=0.055; p=0.056), respectively (Table 5). Multivariate logistic regression confirmed that SIT and OFT independently increased SSI risk, with SIT patients six times more likely [OR 6.0, 95% CI 2.7-13.4; p < 0.001] and OFT patients three times more likely (OR 3.1, 95% CI 1.1-8.4; p = 0.027) to develop infection. Additional independent predictors included wound dehiscence [OR 50.6, 95% CI 28.7-89.5; p < 0.001], being overweight [OR 2.0, 95% CI 1.0-3.5; p=0.037] and undergoing surgery in the North Metropolitan region (OR 2.6, 95% CI 1.3-4.9; p = 0.003). Clear surgical margins were protective, reducing the risk of SSI by 60% (p = 0.002) (Figure 2).

**Table 5:** Univariate analysis – with the likelihood of surgical site infection (SSI) as the dependent variable Values are the number of participants (%) unless otherwise indicated. Pearson Chi-Square analysis, and Fisher’s Exact Test (for cell values <5), and * denotes significance at p<0.05 (indicated in bold) ^a^ BMI: Body mass index; ^b^ IVABs: Intravenous antibiotics; ^c^ SPD: Sacrococcygeal pilonidal sinus disease

Variable (N = 774)		Surgical Site Infection Risk Factors		
		SSI (n)	SSI (%)	p–value
Age	15 to 19 years	39	24.2%	0.133
	20 to 29 years	119	32.1%	
	Older than 30 years	65	26.9%	
Region	SMHS	106	27.9%	0.002*
	EMHS	47	22.3%	
	NMHS	70	38.3%	
Gender	Male	179	29.0%	0.808
	Female	44	28.0%	
BMI^ a^	Healthy weight	50	22.6%	0.055
	Overweight	84	31.6%	
	Obese	89	31.0%	
IVABs on induction ^b^	No	3	20.0%	0.573
	Yes	220	29.0%	
Drain in-situ	No	106	26.0%	0.073
	Yes	117	31.9%	
Query infected site	No	199	27.3%	<0.001*
	Yes	24	53.3%	
Type of skin-prep	Povidone/Iodine	170	26.4%	0.001*
	Chlorhexidine	53	40.8%	
Procedure index	Primary procedure	181	27.5%	0.056
	Re-do procedure	42	36.2%	
Closure technique	Primary closure	194	28.4%	0.447
	Secondary intention	29	32.2%	
Methylene blue	No	180	30.9%	0.024*
	Yes	43	22.4%	
SPD procedure ^c^	KF	96	31.8%	0.002*
	MKF	53	21.9%	
	MLF	22	23.9%	
	SIT	29	32.2%	
	OFT	23	47.9%	
Diabetes	No	189	27.0%	0.001*
	Yes	34	45.3%	
Smoker	No	90	23.7%	0.002*
	Yes	133	33.8%	
Hirsutism	No	131	23.0%	<0.001*
	Yes	92	45.1%	
Wound classification	Clean	21	20.8%	0.140
	Clean/Contaminated	162	29.6%	
	Contaminated	40	32.0%	
Hematoma	No	200	28.2%	0.221
	Yes	23	35.4%	
Dehiscence	No	153	27.6%	0.244
	Yes	70	31.8%	
Seroma	No	220	28.8%	1.000
	Yes	3	27.3%	
Any post-op complication	No	13	2.7%	<0.001*
	Yes	210	72.9%	
Involved margins	No	75	54.7%	<0.001*
	Yes	117	22.7%	

Risk factors for wound dehiscence

The overall rate of WD rate was 28.4% (n = 220). Age was significantly associated with WD, with patients aged 20-29 years (34.6%) and those over 30 years exhibiting higher rates (both 34.6%) than patients aged 15-19 years (22.9%, p = 0.030). BMI demonstrated a strong association with WD, with obese patients experiencing the highest rates (42.2%) compared to overweight (28.9%) and healthy weight individuals (24.0%, p < 0.001). Use of drains was associated with increased WD (36.8% vs 26.8%, p = 0.005), as was surgery in areas of suspected infection (60.9% vs 31.2%, p = 0.003). Chlorhexidine skin preparation was also linked with higher rates of WD than povidone-iodine (45.5% vs 29.3%, p < 0.001).

Procedure type was significant, with KF (37.1%) and OFT (39.6%) associated with higher rates than MKF (25.2%) and MLF (30.4%, p = 0.018). Comorbidities were strongly associated with WD: diabetes (62.3% vs 29.2%, p < 0.001), smoking (38.3% vs 25.7%, p < 0.001), and hirsutism (54.1% vs 24.8%, p < 0.001). Use of methylene blue was associated with lower rates of WD (26.5% vs 34.3%, p = 0.048). WD was strongly associated with SSI (85.1% vs 11.2%, p < 0.001) and with involved surgical margins (60.0% vs 26.6%, p < 0.001). Other variables, including region, gender, intravenous antibiotics, strict bed rest, wound classification, haematoma, and seroma, were not significantly associated with WD. Univariate analysis, with likelihood of WD as the dependent variable, is presented in Table 6.

**Table 6:** Univariate analysis – with likelihood of wound dehiscence (WD) as dependent variable Values are the number of participants (%) unless otherwise indicated. ^*^ Pearson Chi-Square analysis, and Fisher’s Exact Test (for cell values <5), and * denotes significance at p<0.05 (indicated in bold) ^a^ BMI = Body Mass Index, ^b^ IVABs - Intravenous Antibiotics, ^c^ SPD - Sacrococcygeal pilonidal sinus disease

Variable (N = 684)		Wound Dehiscence Risk Factors		
		Dehiscence (n)	Dehiscence (%)	p–value
Age	15 to 19 years	32	22.9%	0.030*
	20 to 29 years	113	34.6%	
	Older than 30 years	75	34.6%	
Region	SMHS	110	33.5%	0.075
	EMHS	48	25.8%	
	NMHS	62	36.5%	
Gender	Male	177	32.8%	0.506
	Female	43	29.9%	
BMI^ a^	Healthy Weight	49	24.0%	<0.001*
	Overweight	68	28.9%	
	Obese	103	42.2%	
IVABs on Induction ^b^	No	2	20.0%	0.514
	Yes	218	32.2%	
Drain in-situ	No	85	26.8%	0.005*
	Yes	135	36.8%	
Query infected site	No	206	31.2%	0.003*
	Yes	14	60.9%	
Type of skin-prep	Povidone/Iodine	165	29.3%	<0.001*
	Chlorhexidine	55	45.5%	
Procedure index	Re-do procedure	35	39.3%	0.121
	Primary procedure	185	31.1%	
Strict Bed rest	No	23	41.1%	0.136
	Yes	197	31.4%	
Methylene Blue	No	170	34.3%	0.048*
	Yes	50	26.5%	
SPD Procedure^ c^	KF	112	37.1%	0.018*
	MKF	61	25.2%	
	MLF	28	30.4%	
	OFT	19	39.6%	
Diabetes	No	182	29.2%	<0.001*
	Yes	38	62.3%	
Smoker	No	86	25.7%	<0.001*
	Yes	134	38.3%	
Hirsutism	No	127	24.8%	<0.001*
	Yes	93	54.1%	
Wound Classification	Clean	24	25.8%	0.053
	Clean/Contaminated	157	31.5%	
	Contaminated	39	41.9%	
Hematoma	No	218	32.1%	0.597
	Yes	2	50.0%	
Surgical Site Infection	No	55	11.2%	<0.001*
	Yes	165	85.1%	
Seroma	No	220	32.4%	0.182
	Yes	0	0.0%	
Any post-op complication	No	0	0.0%	<0.001*
	Yes	220	88.7%	
Involved Margins	No	124	26.6%	<0.001*
	Yes	69	60.0%	

**Figure 3:**
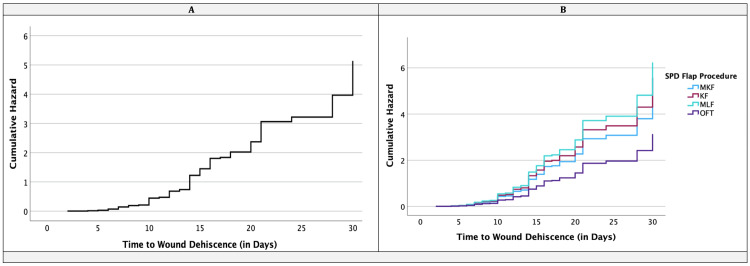
30-day wound dehiscence hazard function curves by SPD flap procedure groups KF: Karydakis flap; MKF: Modified Karydakis flap; MLF: Modified Limberg Flap; OFT: Other flap Techniques Panel A = Hazard Function for Wound Dehiscence at the mean of covariates Panel B = Hazard Function for Wound Dehiscence for SPD Flap Procedures

Cox regression demonstrated that procedure type and smoking status were significant predictors of WD (Table 7; Figure 3). Compared with OFT, KF was associated with a hazard ratio (HR) of 1.78 (95% CI 1.04-3.05, p = 0.037), and MLF with a HR of 1.99 (95% CI 1.06-3.73, p = 0.031). Smoking was also independently associated with WD (HR 1.37, 95% CI 1.02-1.85, p = 0.040). Other covariates, including age, sex, BMI, diabetes, hirsutism, drain use, methylene blue use, redo procedures, and inpatient SSI, were not statistically significant in the Cox model. Hazard plots are shown in Figure 3, with regression results summarised in Table 7.

**Table 7:** Model covariates for wound dehiscence risk factors post definitive SPD procedure ^*^ Bolded denotes significance at p<0.05 Age and BMI are continuous variables ^†^ This parameter is compared to the OFT surgical procedures which is set to zero ^‡^ This parameter is compared to female sex which is set to zero ^§^ This parameter is compared to “no” response which is set to zero ^||^ This parameter is compared to NMHS region which is set to zero ^¶^ This parameter is compared to “yes” response which is set to zero SPD: Sacrococcygeal pilonidal sinus disease; BMI: Body Mass Index; KF: Karydakis Flap; MKF: Modified Karydakis Flap; MLF: Modified Limberg’s Rotational Flap. Due to the low frequency of Bascom’s Cleft Lift procedure (BCL), Gluteus Maximus Myocutaneous Rotational Flap (GRF), Z-plasty Flap (ZP), V-Y Advancement Flap (VY), they were grouped as Other Flap Techniques (OFT)

Variable (N=684)		B	SE	Sig.	Exp(B)	(B) 95.0% CI	
						Lower	Upper
SPD Flap Procedure ^†^	MKF	0.45	0.28	0.111	1.57	0.90	2.73
	KF	0.58	0.28	0.037	1.78	1.04	3.05
	MLF	0.69	0.32	0.031	1.99	1.06	3.73
Age	Years	0.01	0.01	0.554	1.04	0.99	1.02
BMI (kg/m^2^)	nn	0.01	0.01	0.704	1.03	0.99	1.02
Sex ^‡^	Male	0.10	0.19	0.590	1.11	0.77	1.59
Smoker ^§^	Yes	0.31	0.15	0.040	1.37	1.02	1.85
IVABs on Induction ^§^	Yes	0.42	0.74	0.574	1.52	0.36	6.45
Drain In-Situ ^§^	Yes	0.03	0.15	0.857	1.03	0.77	1.37
SSI Index Inpatient ^§^	Yes	0.62	0.68	0.363	1.85	0.49	7.02
Region ^||^	SMHS	0.29	0.18	0.097	1.34	0.95	1.89
	EMHS	0.06	0.21	0.787	1.06	0.70	1.60
Diabetes ^¶^	No	0.05	0.21	0.811	1.05	0.70	1.57
Hirsutism ^¶^	No	0.02	0.16	0.885	1.02	0.75	1.40
Query Infected Site ^¶^	No	0.12	0.30	0.696	1.12	0.63	2.01
Re-Do Procedure ^¶^	No	0.12	0.21	0.576	1.12	0.75	1.70
Hematoma Index Inpatient ^¶^	No	0.20	0.80	0.808	1.22	0.25	5.81

Thirty-day representation and readmission

Within 30 days, 209 patients (27.0%) represented to the hospital, half of whom presented to the emergency department. KF accounted for nearly half of all re-presentations, while OFT had the highest proportional representation rate (45.0%). The most frequent reason for re-presentation was SSI, present in 49.8% of cases, most of which were superficial. Post SPD procedure 30-day re-presentation, destination and reason details are provided in Table 8.

**Table 8:** Post SPD procedure 30-day re-presentation, destination and reason Values are the number of participants (%) unless otherwise indicated. ^* ^Pearson Chi-Square analysis, and Fisher’s Exact Test (for cell values <5), and * denotes significance at p<0.05 (indicated in bold) ^a^ SPD - Sacrococcygeal pilonidal sinus disease, ^b^ ED = Emergency department,^ c^ GP = General Practitioner, ^d^ SSI = Surgical site infection,^ e^ WD = Wound dehiscence

Variable (N=774)	SPD ^a^ Surgery Procedures						
Represented in the 1st month							
	Cohort	KF	MKF	MLF‎	SIT	OFT	p-value
No	565	202	193	70	67	33	0.015*
	73.0%	35.8%	34.2%	12.4%	11.9%	5.8%	
Yes	209	100	49	22	23	15	
	27.0%	47.8%	23.4%	10.5%;	11.0%	7.2%	
Representation destination							
	Cohort	KF	MKF	MLF‎	SIT	OFT	p-value
Emergency department (ED) ^b^	105	50	24	9	13	9	0.034*
	50.2%	47.6%	22.9%	8.6%	12.4%	8.6%	
Ward	9	2	6	1	0	0	
	4.3%	22.2%	66.7%	11.1%	0.0%	0.0%	
Outpatient clinic	66	30	12	12	8	4	
	31.6%	45.5%	18.2%	18.2%	12.1%	6.1%	
General practitioner (GP) ^c^	29	18	7	0	2	2	
	13.9%	62.1%	24.1%	0.0%	6.9%	6.9%	
Representation reason							
	Cohort	KF	MKF	MLF‎	SIT	OFT	p-value
Pain	8	2	3	1	3	0	<0.001*
	4.3%	22.2%	33.3%	11.1%	33.3%	0.0%	
SSI ^d^	104	64	22	5	7	6	
	49.8%	61.5%	21.2%	4.8%	6.7%	5.8%	
WD ^e^	70	27	19	16	0	8	
	33.5%	38.6%	27.1%	22.9%	0.0%	11.4%	
Pain & SSI	12	4	5	0	2	1	
	5.7%	33.3%	41.7%	0.0%	16.7%	8.3%	
High output drain‎ or Vac issues	5	0	0	0	5	0	
	2.4%	0.0%	0.0%	0.0%	100.0%	0.0%	
Tertiary bleeding	6	2	0	0	4	0	
	2.9%	33.3%	0.0%	0.0%	66.7%	0.0%	
Hypergranulation	2	0	0	0	2	0	
	1.0%	0.0%	0.0%	0.0%	100.0%	0.0%	
Constipation	1	1	0	0	0	0	
	0.5%	100.0%	0.0%	0.0%	0.0%	0.0%	

Thirty-day readmission occurred in 58 patients (7.5%). KF and MKF together accounted for nearly three-quarters of these readmissions. SSI (34.5%) and WD (31.0%) were the leading causes, while refashioning of wounds was rare. Vacuum-assisted devices used in SIT procedures also contributed to readmission, mainly due to persistently high drainage outputs. No deaths occurred during the follow-up period (Table 9).

**Table 9:** Post SPD Procedure SPD 30-day readmission and reason Values are the number of participants (%) unless otherwise indicated. ^*^ Pearson Chi-Square analysis, and Fisher’s Exact Test (for cell values <5), and * denotes significance at p<0.05 (indicated in bold) ^a^ SPD: Sacrococcygeal pilonidal sinus disease, ^b^ SSI: Surgical site infection,^ c^ WD: Wound dehiscence, ^d^ Vac: Negative pressure wound dressing system

Variable (N=774)	SPD ^a^ Surgery Procedures						
Readmitted 1st Month							
	Total cohort	KF	MKF	MLF‎	SIT	OFT	p-value
No	716	282	228	87	76	43	0.027*
	92.5%	39.4%	31.8%	12.2%	10.6%	6.0%	
Yes	58	20	14	5	14	5	
	7.5%	34.5%	24.1%	8.6%	24.1%	8.6%	
Readmission Reason							
	Sub-group	KF	MKF	MLF‎	SIT	OFT	p-value
Pain	2	2	0	0	0	0	0.029*
	3.4%	100.0%	0.0%	0.0%	0.0%	0.0%	
SSI ^b^	20	8	5	2	3	2	
	34.5%	40.0%	25.0%	10.0%	15.0%	10.0%	
WD ^c^	18	8	4	2	1	3	
	31.0%	44.4%	22.2%	11.1%	5.6%	16.7%	
Pain & SSI ^b^	8	2	4	0	2	0	
	13.8%	25.0%	50.0%	0.0%	25.0%	0.0%	
High output drain‎ or Vac ^d^	9	0	1	1	7	0	
	15.5%	0.0%	11.1%	11.1%	77.8%	0.0%	
Refashion wound	1	0	0	0	1	0	
	1.7%	0.0%	0.0%	0.0%	100.0%	0.0%	

## Discussion

This multi-centre cohort study is the first in Australia to evaluate surgical site complications after definitive surgery for sacrococcygeal pilonidal sinus disease incorporating both flap-based and secondary intention techniques. The study focused on SSI and WD, the most common and clinically significant complications of pilonidal surgery [12-14,16,22]. Together they account for the bulk of postoperative morbidity, contribute to hospital re-presentations and readmissions, and impose substantial demands on outpatient wound care services [10,16,18,20,21,23-26]. Their prevention therefore represents a critical benchmark for surgical quality in this field, particularly given the variability in elective surgical approaches for SPD, resulting in significantly different patient outcomes [27,28].

The overall SSI rate of 28.8% and WD rate of 28.4% observed in this cohort are striking, even when compared with the wide ranges reported internationally [10,11,16]. These complication rates are not trivial; both SSI and WD can delay healing by weeks or months, prolonging the requirement for dressings and hospital-in-the-home services, and significantly impact patient wellbeing and productivity. In the context of SPD, where the disease predominantly affects young working adults, the socioeconomic burden of prolonged recovery is considerable. The strong correlation between SSI and WD demonstrated in this study further underscores their clinical importance, as infection and breakdown often coexist, compounding morbidity.

This analysis identified both patient-level and procedural determinants of SSI and WD. On multivariate modelling, secondary intention and other complex flap techniques were independently associated with higher SSI rates, with SIT conferring a six-fold risk and OFT a three-fold risk compared with MKF. WD, meanwhile, was most strongly associated with KF and MLF procedures, as confirmed by Cox regression (Table 7; Figure 3). Patient-related factors also exerted a significant influence. Obesity, diabetes, smoking and hirsutism emerged as consistent predictors of both SSI and WD, highlighting the contribution of systemic and local host factors to wound healing. The interplay between infection, involved surgical margins and dehiscence further reinforces the concept that meticulous operative technique and patient optimisation are crucial in reducing surgical site occurrences.

The importance of surgical margins warrants particular attention. Incomplete excision was associated with higher rates of both SSI and WD, while clear margins were protective. This finding aligns with surgical principles but is particularly relevant in SPD, where disease extent is sometimes difficult to define intraoperatively. Ensuring wide and complete resection may be as important as the choice of closure technique in preventing complications. Similarly, the protective association observed with methylene blue staining suggests that adjunctive measures to aid visualisation of sinus tracts may improve surgical completeness and reduce postoperative morbidity.

Among the procedures compared, the MKF consistently demonstrated the most favourable outcomes across both SSI and WD, supporting its role as the preferred option for elective definitive surgery. However, it should be recognised that even MKF was associated with clinically significant complication rates. Conversely, secondary intention techniques were associated with unacceptably high rates of SSI and re-presentation, yet they remain a pragmatic option when infection precludes safe primary closure. These findings reinforce the principle that no single procedure is universally applicable; rather, outcomes are optimised when patient risk factors, disease complexity and intraoperative findings are integrated into a shared decision-making framework.

Readmissions and re-presentations provide an important measure of the broader healthcare impact of SPD surgery, and in this study, they were predominantly driven by wound-related complications. Within 30 days, 27% of patients re-presented, most often to the emergency department or outpatient clinics, and 7.5% required readmission. SSI accounted for more than one-third of readmissions, while WD contributed to nearly one-third, with a substantial overlap between the two. Secondary intention techniques were associated with the highest readmission rates, whereas modified flap procedures, particularly the MKF, had comparatively lower but still clinically significant representation rates. These findings underline that SSI and WD are not only immediate postoperative complications but also major drivers of hospital resource use, influencing both early re-presentations and readmissions.

This study has several limitations. Its retrospective design relied on administrative and clinical records, with potential for data entry errors and incomplete documentation. Surgical technique was heterogeneous, with variation in flap design, closure method, drain use and perioperative management not consistently captured, limiting the precision of comparisons between techniques. Some procedures, particularly those grouped as other flap techniques, were under-represented, preventing analysis of individual outcomes for Bascom cleft lift, gluteus maximus myocutaneous flap, Z-plasty and V-Y advancement. While data were collected over a prolonged period, extended follow-up was not available for all patients, and recurrence data were incomplete, with late complications possibly under-estimated if patients presented outside the study hospitals. Patient-reported outcomes such as pain, cosmesis and quality of life were not available. Finally, findings are limited to the public healthcare system, which may not reflect private practice. However, review of the complete state data linkage record, including both public and private hospitals, confirmed that more than 85% of elective SPD procedures were conducted in the public sector, suggesting that the findings are broadly representative of overall practice.

Despite these limitations, the study provides important insights. SSI and WD remain common and burdensome complications after SPD surgery, with both patient-level and procedural factors influencing risk. Modified flap procedures, particularly the MKF, offer the most balanced outcomes but do not eliminate the problem. These findings highlight the need for structured training and upskilling to improve operative consistency, optimisation of modifiable patient factors, and continued refinement of surgical techniques. Future prospective studies should place SSI and WD at the centre of outcome reporting, with longer follow-up and inclusion of patient-centred metrics to fully capture the burden of these complications.

The principal strength of this study lies in its comprehensive representation of contemporary surgical practice, combined with long-term follow-up of all patients to capture genuine long-term outcomes. Recognising that some patients may not return to their index centre for complication management, as reported in previous studies, this study employed triangulation of follow-up data across multiple sources to achieve a more accurate and complete depiction of outcomes.

## Conclusions

SSI and WD are the most frequent and burdensome complications following definitive surgery for sacrococcygeal pilonidal disease. This study identified key patient-level risk factors, including obesity, diabetes, smoking and hirsutism, as well as procedural determinants such as incomplete margins and closure technique. Clear margins were protective, and the MKF demonstrated the most favourable overall risk profile, whereas secondary intention and other complex flaps were associated with higher complication rates. SSI and WD were also the predominant causes of 30-day re-presentations and readmissions, confirming their central role in hospital utilisation and patient morbidity. These findings reinforce the importance of optimising modifiable risk factors, refining operative techniques, and promoting structured training to reduce variation in practice. Future prospective studies should prioritise SSI and WD as key outcomes, with long-term follow-up to define their contribution to recurrence and the overall healthcare burden.
